# Succession in the Gut Microbiome following Antibiotic and Antibody Therapies for *Clostridium difficile*

**DOI:** 10.1371/journal.pone.0046966

**Published:** 2012-10-10

**Authors:** Gregory L. Peterfreund, Lee E. Vandivier, Rohini Sinha, Andre J. Marozsan, William C. Olson, Jun Zhu, Frederic D. Bushman

**Affiliations:** 1 Department of Microbiology, Perelman School of Medicine at the University of Pennsylvania, Philadelphia, Pennsylvania, United States of America; 2 Department of Genetics, Perelman School of Medicine at the University of Pennsylvania, Philadelphia, Pennsylvania, United States of America; 3 Progenics Pharmaceuticals, Tarrytown, New York, United States of America; Institut Pasteur, France

## Abstract

Antibiotic disruption of the intestinal microbiota may cause susceptibility to pathogens that is resolved by progressive bacterial outgrowth and colonization. Succession is central to ecological theory but not widely documented in studies of the vertebrate microbiome. Here, we study succession in the hamster gut after treatment with antibiotics and exposure to *Clostridium difficile*. *C. difficile* infection is typically lethal in hamsters, but protection can be conferred with neutralizing antibodies against the A and B toxins. We compare treatment with neutralizing monoclonal antibodies (mAb) to treatment with vancomycin, which prolongs the lives of animals but ultimately fails to protect them from death. We carried out longitudinal deep sequencing analysis and found distinctive waves of succession associated with each form of treatment. Clindamycin sensitization prior to infection was associated with the temporary suppression of the previously dominant *Bacteroidales* and the fungus *Saccinobaculus* in favor of *Proteobacteria*. In mAb-treated animals, *C. difficile* proliferated before joining *Proteobacteria* in giving way to re-expanding *Bacteroidales* and the fungus *Wickerhamomyces*. However, the *Bacteroidales* lineages returning by day 7 were different from those that were present initially, and they persisted for the duration of the experiment. Animals treated with vancomycin showed a different set of late-stage lineages that were dominated by *Proteobacteria* as well as increased disparity between the tissue-associated and luminal cecal communities. The control animals showed no change in their gut microbiota. These data thus suggest different patterns of ecological succession following antibiotic treatment and *C. difficile* infection.

## Introduction

The importance of an intact gut microbiota to the host has been recognized since at least the 1950s [1], but only recently have the profound effects of host-microbiome interactions been appreciated. The gut microbiota is involved in functions as diverse as promoting digestion and metabolism of food [2], synthesis of essential vitamins [3], and stimulation and regulation of the host immune system. Endogenous microbial inhabitants provide colonization resistance to infection by other microbes, including cholera [4], salmonella [5], [6], streptococcus [7], and most notably *Clostridium difficile*. In multiple animal models [8], [9] and clinically in humans [10], exposure to antibiotics is the strongest predictor of *C. difficile*-associated diarrhea (CDAD), presumably resulting from a disruption of the established microbial community.

*C. difficile* is a Gram-positive, sporulating, anaerobic Firmicute. A hypervirulent strain has been associated with an intensifying worldwide epidemic of nosocomial disease, including profuse diarrhea, pseudomembranous typhlocolitis, multiple organ dysfunction syndrome, and death. A recent analysis found 336,000 *C. difficile*-related hospitalizations in the US in 2009, which account for nearly 1% of the total hospitalizations and encompass a 300% increase since 1993 [11]. For patients with CDAD as their primary diagnosis, the mean cost and length of hospital stay were $10,100 and 6.9 days, respectively.

Pathogenic strains of *C. difficile* typically produce two main exotoxins, Clostridial cytotoxins A and B, which cause most of the morbidity of CDAD and are attractive targets for inhibition as resistance to disease correlates with serum anti-toxin antibodies in man [12], [13], [14], and presence of toxin in serum has been correlated with severity of disease in anizmal models [15].

Until recently, the best available treatments for CDAD were the antibiotics vancomycin and metronidazole. These represent a double-edged sword in that they suppress *C. difficile*, but they, in turn, disrupt the microbiota, potentially prolonging susceptibility beyond the end of therapy. A recently-approved narrow-spectrum antibiotic against *C. difficile* could potentially reduce microbiota disruption as well as recurrence in the course of therapy [16]. Two promising alternative therapies are treatment with exogenous monoclonal antibody (mAb) to neutralize the toxin, and fecal microbial transplant to reconstitute the gut microbiota with a healthy donor community, which should have minimal or beneficial effects on the recipient's intestinal community [17], [18], [19]. The mechanism of action by which the microbiota suppress disease is poorly understood. Proposed inhibitory factors include faster replication, competition for mucin degradation products, occupation of binding sites, neutralization of toxin activity, or direct inhibition through toxic metabolites or bacteriocins [9]. Elucidating these mechanisms may be key to the potential development of safe and effective therapies to treat *C. difficile* infection.

In order to investigate the roles of the normal intestinal microbiota in preventing establishment of *C. difficile* in the gut and in modulating response to CDAD therapy, a longitudinal study of *C. difficile* infection and subsequent treatment in hamsters was performed.

## Results

### Survival outcomes

**Figure 1:**
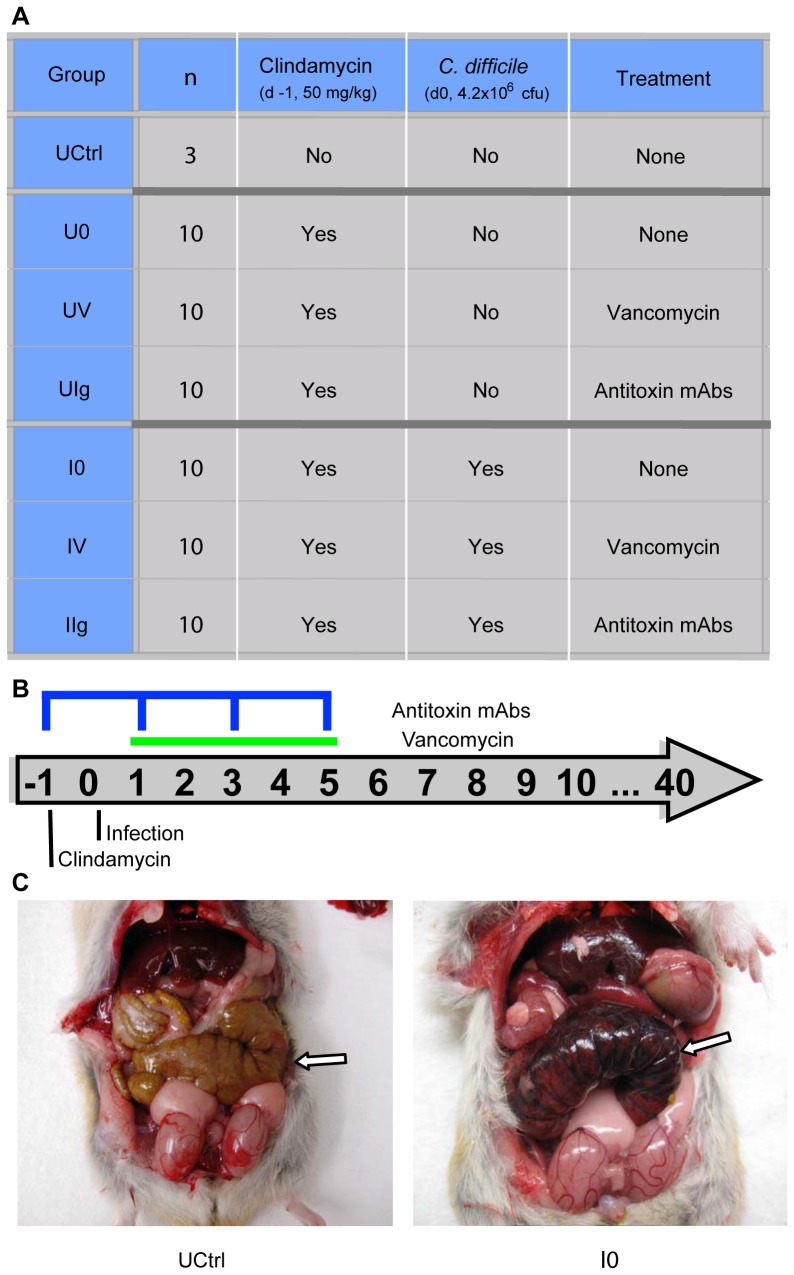
Experimental design and pathology. (A) Hamsters were divided into seven groups, corresponding to one untreated control group (n = 3) and two clindamycin-treated arms with and without *C. difficile* challenge. Within each arm, hamsters were divided into three treatment groups- none, vancomycin, and antitoxin monoclonal antibody (n = 10 per treatment group). (B) Timeline of interventions. Clindamycin, *C. difficile*, vancomycin (green bar), and mAb (blue indicator) were given to the designated groups on the days indicated. (C) Gross pathology of Golden Syrian hamsters. Arrow indicates the cecum. [Left] Untreated control (UCtrl) animal on day 40; [Right] Infected, no treatment (I0) animal on day 2. The cecum of the infected, untreated hamster is enlarged, hyperemic, inflamed, and partially necrotic.

The study was designed to include an untreated control (UCtrl) group of hamsters that received no intervention, and two arms that received clindamycin either with (infected, I) or without (uninfected, U) inoculation with *C. difficile*. In each of those arms, hamsters were assigned to groups to which no therapy (groups I0 and U0), vancomycin treatment (groups IV and UV), or an experimental antitoxin antibody therapy (groups IIg and UIg) was administered. The groups are summarized in Figure 1A, and the timing of treatment is shown in Figure 1B and described in the Materials and Methods (Study design). Figure 1C shows the cecum of a representative hamster in the uninfected control (UCtrl) group on day 40 of the study. Figure 1D shows the cecum of a representative hamster in the infected, untreated (IO) group on day 2 of the study.

Despite rigorous contamination controls, including separate rooms and caretakers for infected and uninfected groups, as well as decontamination footbaths, this study was complicated by the development of CDAD in all animals that had received clindamycin. *C. difficile* recovered from the stool samples of the clindamycin-treated animals was found to be ribotype 12 (data not shown), which is the same ribotype as that of the ATCC 43596 strain of *C. difficile* that was used to infect the study animals. Because ribotype 12 is an uncommon ribotype/strain of *C. difficile*, it is highly likely that the control animals were infected due to contamination of the study facility by the ATCC 43596 infection strain [20].

**Figure 2:**
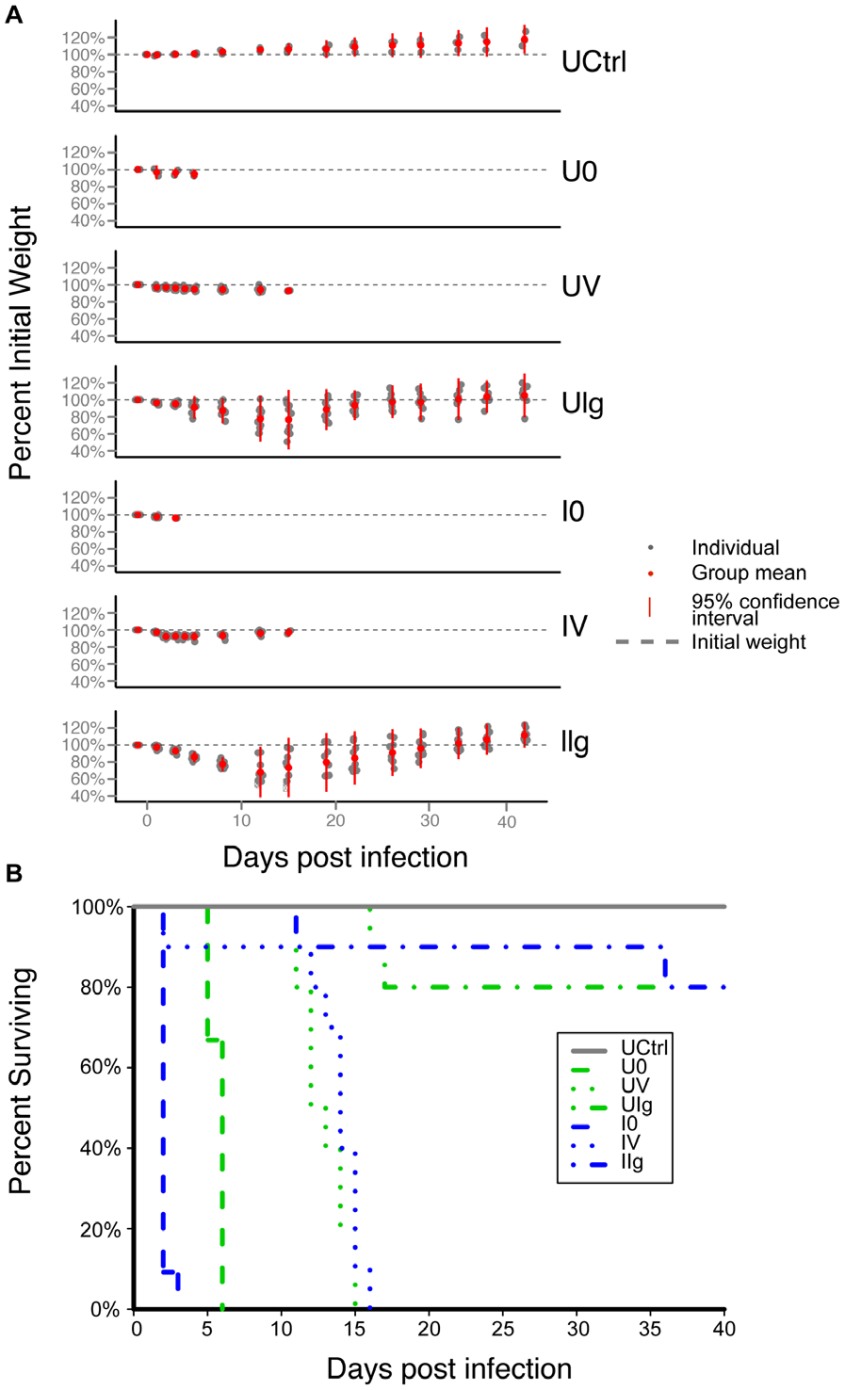
Natural history of infection includes weight loss and high mortality rate. (A) Mean change in percent weight per group over the course of the experiment. Deceased hamsters were dropped from the plot. Red dot- mean weight as percent of initial; gray dot- individual weight as percent of initial; red bar- range within 2 standard deviations of the mean; gray dashed line- original weight. (B) Kaplan-Meier survival curve. Untreated control (UCtrl) hamsters survived the duration of the study. Both infected and uninfected untreated animals showed 100% mortality within six days. One animal died in both vancomycin-treated groups on day 2, while the rest of the animals survived between 11 and 16 days post infection, approximately one week after cessation of treatment. Two animals died in the uninfected, mAb-treated group, and one animal died in the infected, mAb-treated group about two weeks post infection. One more hamster from the infected, mAb-treated group died shortly before the end of the experiment and the rest survived until they were euthanized on day 40.

Seven of the untreated control group (UCtrl) animals were sacrificed at the start of the study (day 0) as per the protocol, The remaining three animals survived to the end of the study and gained about 20% more weight compared to their weight at the start of the study (Figure 2A). In the group that was actively infected with *C. difficile* without further therapy (I0), four hamsters had died and five others were moribund by day 2.

In the uninfected/untreated group (U0), seven animals were terminated on day 2 (n = 6) or day 3 (n = 1) in order to provide time-matched microbiota controls for the infected/untreated group (I0). Of these seven animals in the U0 group, all were found to have unremarkable ceca, with the exception of a single animal that was observed to be moribund on day 2 with an inflamed cecum indicative of CDAD. Three additional hamsters in the U0 group were not subject to matched euthanasia, and these animals were found moribund or dead on days 5 and 6 with inflamed ceca and minimal weight loss (Figure 2A). These outcomes were unexpected and may also be a result of the above-mentioned general contamination of the study facility by the *C. difficile* strain used in the study.

The percent survival of hamsters in the different study groups is shown in Figure 2B. Of the vancomycin-treated hamsters (groups UV and IV), one animal from each group was found dead on day 2, while the rest of the animals in these groups died between days 11 and 16 of fulminant CDI that is characteristic of this model, despite maintaining their initial weight. Thus, vancomycin-treated animals survived longer, but were not protected from death.

In the uninfected and infected mAb-treated groups (UIg and IIg), most of the animals lost weight by days 5–7, but then eventually recovered and increased in body weight (Figure 2A). The infected mAb-treated group lost two out of ten hamsters on days 11 and 36. Neither of the animals that had died showed gross pathology consistent with CDAD. The animals in the UIg- and IIg-treated groups had lost 25% of their initial weight (greater than any of the vancomycin hamsters) and 4% of their initial weight, respectively. Two animals in the uninfected group died on days 16 and 17 (Fig. 2B); the former with a 49 g weight loss and a discolored cecum and the latter with a 54 g weight loss and a normal-appearing cecum. The cause(s) of death of these particular animals is/are uncertain.

For the microbiota analysis, we retained samples from the untreated control (UCtrl), vancomycin-treated (IV) and mAb-treated groups (IIg and UIg, Figure 1A), which allowed comparison of the results obtained from animals undergoing different therapies and the potential effects of longitudinal changes in gut microbial communities.

### Longitudinal analysis of *C. difficile* infection using quantitative PCR

**Figure 3:**
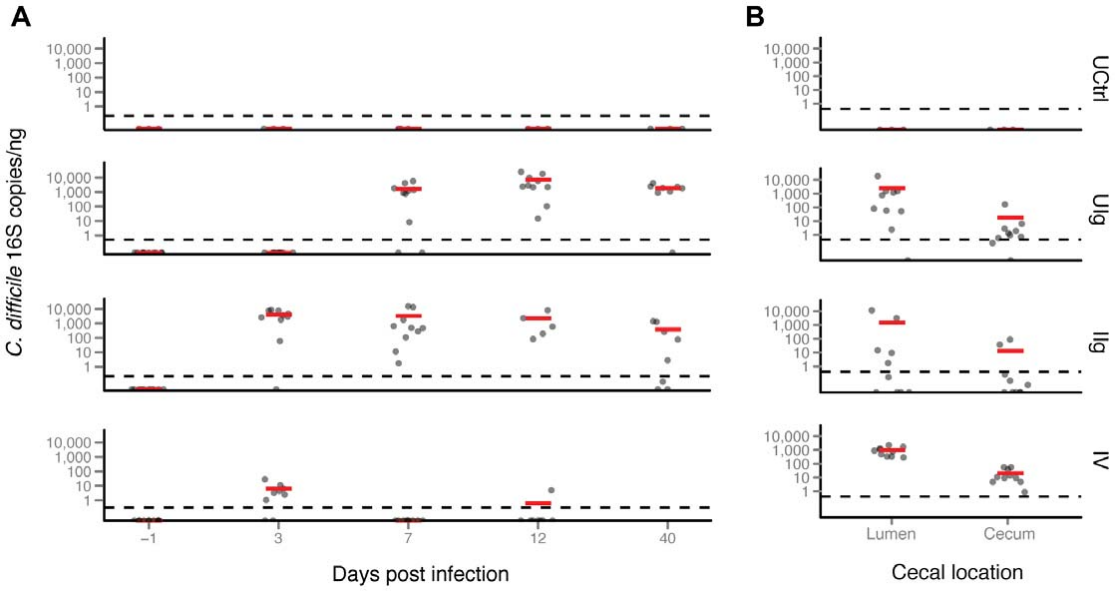
*C. difficile*-specific Taqman qPCR analysis of hamster feces and ceca. (A) A custom qPCR specific for *C. difficile* DNA was consistently negative for all tested samples from the untreated control group of hamsters. *C. difficile* DNA in the UIg group rises above the limit of detection by day 7 and remains elevated. The infected hamsters have a detectable spike by day 3 despite having no detectable *C. difficile* initially. The mAb-treated groups have near-complete survival to day 40 despite having a significant pathogen DNA burden as determined by PCR analysis. Vancomycin-treated animals have a lower spike by day 3 that falls below the limit of detection for most of the subsequent samples. (B) *C. difficile* 16S RNA copies were detected in luminal and cecal samples. Red bar- group mean 16S copies/ng DNA; gray dots- individual mean 16S copies/ng DNA. Limit of detection is 27 copies per reaction, or ca. 7.9×10^3^ *C. difficile* genome equivalents per gram of dry weight feces assuming perfect recovery.

A *C. difficile* 16S rRNA gene-specific Taqman qPCR was developed as a means of tracking the *C. difficile* infection over the course of the experiment (Figure 3 A and B). None of the stool or cecal samples tested from the untreated control group contained detectable *C. difficile* DNA. This group was likely exposed to *C. difficile*, but the inoculum was not able to colonize at a high enough level to detect. In this study, all clindamycin-treated animals became positive for *C. difficile*. The UIg group showed no *C. difficile* DNA in the early time points, but acquired a large *C. difficile* DNA population by day 7 that was maintained until the end of the study. The IIg group did not contain *C. difficile* on day -1 but showed a mean of 7,336 copies per ng of *C. difficile* DNA by day 3. This high level drifted downward to 710 copies per ng DNA, but remained positive over the remainder of the study. Similarly, the IV group had no detectable *C. difficile* initially, but showed 8.3 copies per ng DNA by day 3. *C. difficile* DNA content was found to be below the limit of detection in the remaining fecal samples, except for one animal on day 12. These observations parallel those seen in the 16S rRNA gene sequencing, with the highest abundance of *C. difficile* in the infected groups (discussed below) and with resolution to a lower fraction of the population by day 40. By the last time point, most hamsters that had been treated with clindamycin or with the anti-toxin mAbs remained PCR-positive for *C. difficile*, although the *C. difficile* DNA concentrations were decreased about ten-fold from the peak.

The cecal, luminal and tissue-associated samples from the untreated controls had no detectable *C. difficile* DNA. The three other groupsthat had received clindamycin treatment, i.e., UIg, IIg and IV had mean luminal concentrations of *C. difficile* DNA of 2200, 1500, and 940 copies/ng, respectively, and mean tissue-adherent concentrations of *C. difficile* DNA of 17, 12, and 20 copies/ng, respectively. The lower number of copies of *C. difficile* DNA per ng in the tissue-associated samples is likely due to a greater admixture of hamster DNA, although another possibility is that *C. difficile* grew out between the time of the last sample and the death of the animals.

Cecal and fecal levels of vegetative *C. difficile* were determined at or near termination for six hamsters selected at random in each of groups I0, IV and IIg. Cecal counts were highest in the I0 group (8.98±0.34 log_10_ colony-forming units, CFU), intermediate in the IV group (6.59±0.92 log_10_ CFU) and lowest in the IIg group (3.39±1.30 log_10_ CFU). Fecal counts followed a similar trend with the exception that levels were undetectable when feces were obtained 1–2 days prior to termination in three animals in the IV group.

### Initial microbiome composition

To characterize the gut bacterial communities and longitudinal changes with in the different treatment groups, we used 454 pyrosequencing of 16S rRNA gene tags. Stool was recovered, DNA extracted, and samples PCR-amplified using primers that bind to the V1V2 region of the bacterial 16S rDNA. Each primer was barcoded to allow pyrosequencing of amplicons in pools, and subsequent assignment of reads to samples using the barcode information. Reads were processed using the QIIME pipeline [21], in which reads were grouped into representative operational taxonomic units (OTUs) at 97% identity, taxonomy assigned using RDP and beta diversity compared using UniFrac [21], [22], [23], [24]. A total of 392 stool samples were characterized.

Two types of controls were carried out to monitor contamination during sample preparation. Extraction controls, 36 in total, were carried out in which DNA was purified from stool stabilization buffer, worked up, and sequenced with experimental samples. We also ran 20 PCR controls, in which DNA-free water was PCR amplified and sequenced. All controls were sequenced, even though none showed bands visible by gel electrophoresis. Of 56 controls, 47 did not return more than 200 quality-filtered reads. Two controls had evidence of spillover during extraction, and resembled experimental samples. The remainder were distinguishable by high representation of *Enterobacteriales* and *Campylobacteriales* and thus differed from experimental samples.

**Figure 4:**
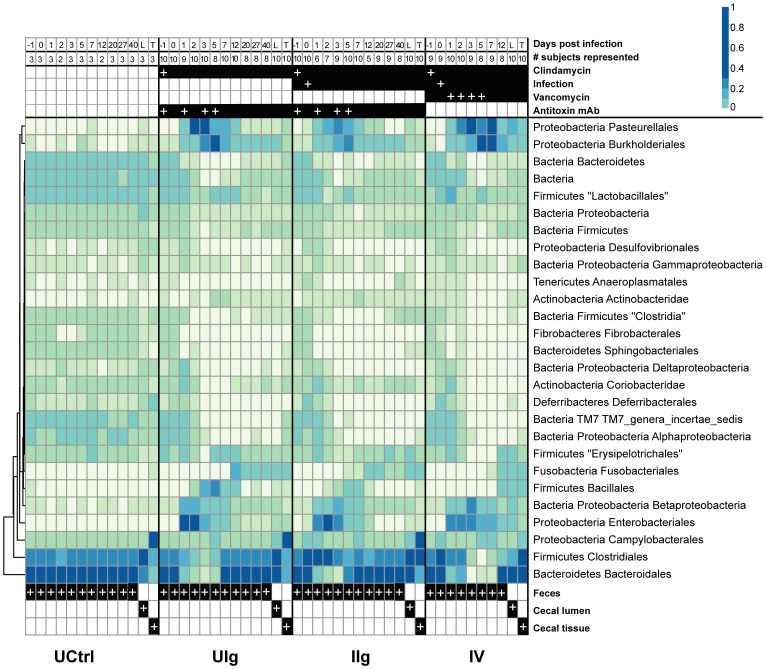
Taxonomic heatmap of fecal and cecal bacterial communities demonstrating large-scale community shift and multiple waves of succession following clindamycin administration. Heatmap showing relative abundance of taxa as a percent of total 16S rRNA tag reads. Each column represents the read-aggregated mean community for that treatment group and day, each row represents a taxon. The uninfected, clindamycin-naïve group is mostly stable and homogenous for the entirety of the experiment, while clindamycin induces a large-scale perturbation in the microbiota. The numbers of animals and the treatments are shown at the top (white plus (“+”) signs indicate dates of administration) and sample origin at the bottom. Proportion contributed by each taxon is shown by the color scale at the top right.

On day -1, animals in all of the study groups harbored similar microbial communities composed primarily of *Bacteroidales* and *Clostridiales* species with smaller contributions by members of the groups *Desulfovibrionales*, *Gammaproteobacteria, Anaeroplasmatales, Actinobacteridae*, and *Fibrobacterales* (Figure 4). Similarity may be due to animals originating from the same vendor and being of similar sex, weight, and age at the beginning of the study, and possibly may also be related to a population bottleneck which led to low genetic diversity among hamsters [25]. Kinship among animals was unknown.

### Effects of the treatments studied on the stool microbiome

**Figure 5:**
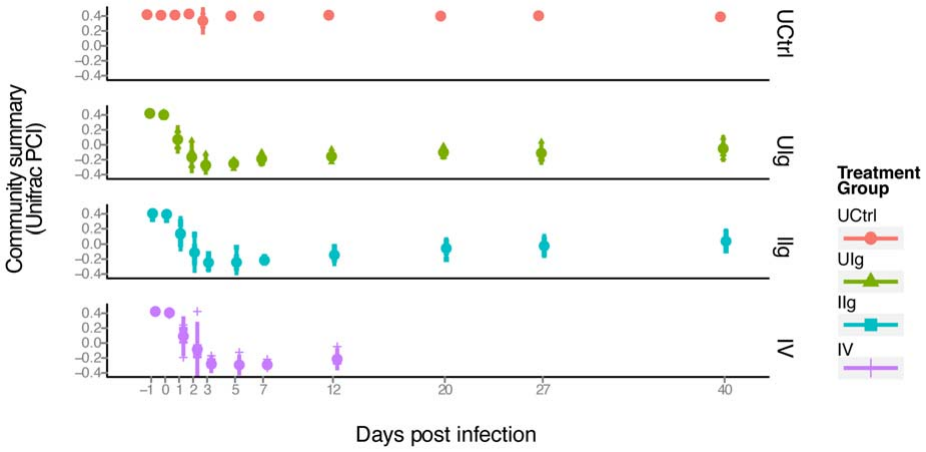
PCoA analysis displaying the community structure through clindamycin treatment and recovery. The x-axis indicates time, the y-axis indicates the value of the first principal coordinate, a summary of the UniFrac matrix of distances between bacterial communities. The color code for the different groups is shown to the lower right. Each point represents the community of one animal on one day and the large colored dot on each day is the mean. Red dots- untreated control group; green triangles- UIg; teal squares- IIg; purple plus signs- IV. Vertical bar- range of two standard deviations from the mean.

To assess changes in community structure, we performed a UniFrac analysis of the sequencing results [24]. UniFrac calculates distances between paired samples based on shared branch length when constituent bacteria are placed onto a phylogenetic tree. The resulting distance matrix of pairwise distances can then be used for a principle coordinates analysis to cluster and visualize samples by similarity. In Figure 5, the first principal coordinate is plotted on the y-axis, which summarizes bacterial community structure, versus time, which is plotted on the x-axis. Separate plots are shown for four treatment groups (UCtrl, UIg, IIg, and IV).

All samples were tightly grouped in the beginning, indicating initially similar communities on days -1 and 0 (value of about 0.4 on the y-axis, Figure 5). The control animals (UCtrl) remained in this state and did not change over the course of the experiment. A rapid population change is visible on days 1 and 2 in the samples from clindamycin-treated animals (Groups UIg, IIg, and IV) as is observed in the change in the y-value from 0.4 to -0.3. These samples remain similar on days 2 and 3. Communities evolve from days 5 to 12, and then reach a stable new state from days 27 to 40, with a y-axis value of about 0.

The effect of clindamycin was profound, with broad reduction in abundance of *Bacteroidetes* and *Firmicutes* (Figure 4). This effect was expected from the known activity of clindamycin against anaerobes and Gram-positive bacteria [26]. Some taxa that were expected to be largely resistant were also suppressed, such as *Gammaproteobacteria*. Concomitantly, previously minor taxa, including species in Proteobacterial orders *Pasteurellales, Enterobacteriales*, and *Burkholderiales*, became the major components of the community from day 1 through day 5. The inoculation with *C. difficile* was associated with a large spike in *Clostridiales* on days 1 and 2 with a smaller increase in *Burkholderiales* and *Pasteurellales* species. Vancomycin treatment prolonged the general effects of clindamycin, notably extending the knockdown of members of *Bacteroidales* and *Clostridiales*.

**Figure 6:**
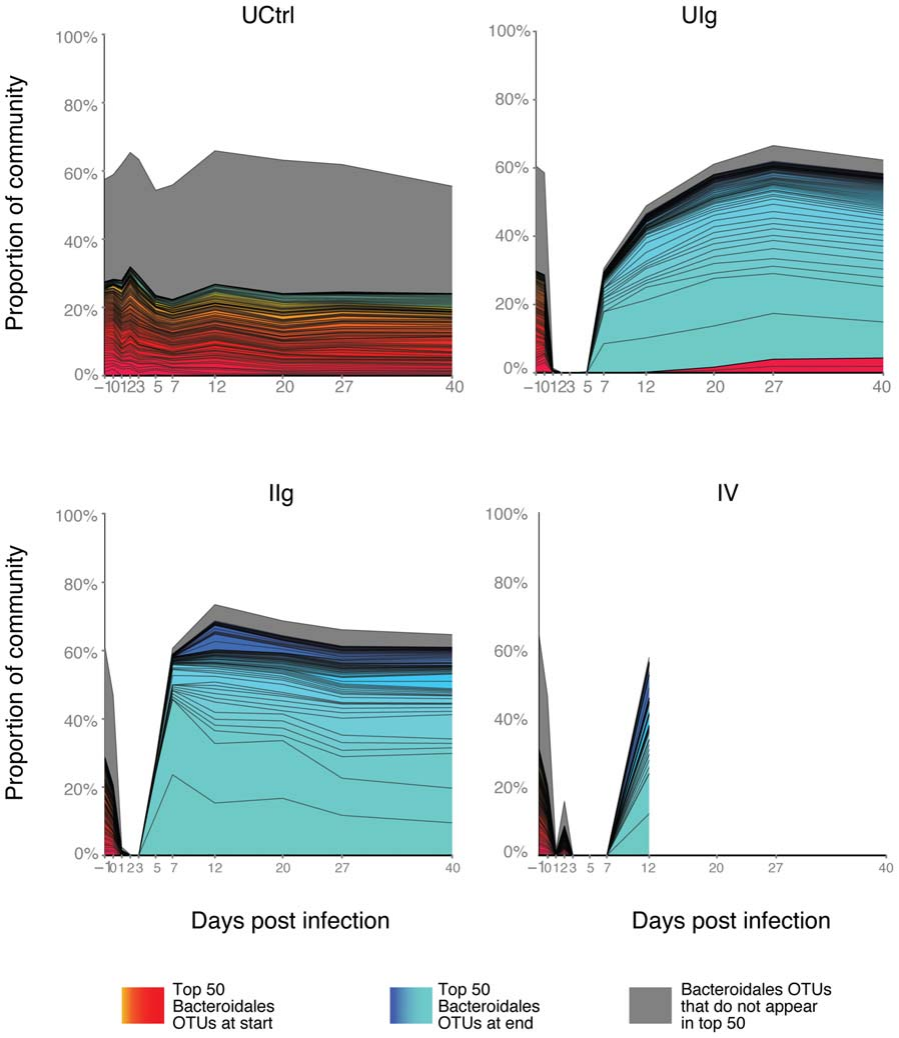
Divergent Bacteroidales present in the hamster gut at the beginning and end of the study. The 50 most abundant Bacteroidales OTUs from each group were identified at the beginning and the end of the study and marked–red indicates Bacteroidales OTUs predominant at the beginning, blue indicates the predominant Bacteroidales at the end of the study, grey indicates remaining, lower abundance Bacteroidales. The x-axis shows time, and the y-axis shows proportion over all bacterial lineages. OTU – color correspondence is constant for all panels.

We found, unexpectedly, that the *Bacteroidales* OTUs observed at the end of the study period were distinct from those present at the beginning (Figure 6). This is illustrated by comparing the most abundant 50 *Bacteroidales* OTUs from days -1 (red) and 40 (blue). For the uninfected, clindamycin-naïve group, the top OTUs comprise about 22% of the community and contained the same lineages over the full time course (red throughout). In the clindamycin-treated individuals, the top OTUs also represent 20–25% of the community, but these rapidly disappeared by day two and did not recover. By contrast, the top 50 OTUs from day 40 begin to expand around day five and eventually represent about 60% of the community. The effect of vancomycin can be seen by the slight delay in the reconstitution of the microbiota relative to the other clindamycin groups immediately before death of the vancomycin treated animals. Thus in clindamycin-treated animals, the stable *Bacteroidales* community present at the end of the study differed from that at the beginning.

The additional effect of *C. difficile* infection on the rest of the microbiota was modest. There was a large increase in *Clostridiales* from days 0 to 2, part of which corresponds to the inoculum. However, the high rate of *C. difficile* viability loss in the initial stages of infection suggests that 10% of the inoculum or less would reach the cecum, and the remaining 4.2×10^5^ cells would be insufficient to directly alter the distribution of the microbiota in a significant manner [27]. As the total gut bacterial population did not decline significantly in previous research on the effects of antibiotics, this enrichment of *Clostridiales*, of which 95–100% is *C. difficile* must therefore correspond to active replication and outgrowth of *C. difficile* in hamsters' intestines. Subtracting *C. difficile* from the community removes much of the consistent differences between the UIg group and the IIg group. This is likely because in this study, all clindamycin-treated animals became infected with *C. difficile*, which had no direct effects on the microbiota.

Vancomycin treatment prolonged the survival of the hamsters, but had lesser effects on the microbiota beyond those associated with clindamycin treatment. The shift to a predominantly *Clostridiales* community in the animals of the IIg group after infection is greatly reduced while the expansion of the *Proteobacteria* species associated with the clindamycin treatment was exaggerated and extended out to at least day 7 in the animals of group IV compared to the animals in the mAb-treated groups UIg and IIg. Vancomycin also prevented a *Bacillales* bloom seen in the other groups on day 2, as well as what appears to be an effect on *Coriobacteridae* and *Erysipelotrichales* (Figure 4).

### Effects of the treatments studied on bacterial communities of the cecal tissue and lumen

We next investigated the microbiota of the cecum, which is a major site of *C. difficile*-induced inflammation (Figure 1C). DNA was extracted from samples taken from the luminal contents and washed cecal tissue of animals in groups UCtrl, UIg, IIg, and IV.. Bacterial 16S rRNA V1V2 sequences were amplified, pyrosequenced, and analyzed as above (Figure 4, columns labeled at the bottom of the figure).

The starting communities in the ceca differed from those in stool. The tissue-associated communities were strongly enriched for three OTUs of *Helicobacter* (each p<1×10^−11^), while the luminal communities contained primarily *Lachnospiraceae* and *Bacteroidales*. Several *Mucispirillum* OTUs trended towards, but did not achieve, significant enrichment in the tissue community. Stool, in contrast, was dominated by *Bacteroidales* alone.

Following clindamycin treatment, the tissue-associated communities showed only modest changes in the antibody treated groups (IIg and UIg) at the end of the 40-day experiment. The luminal communities in the animals of groups IIg and UIg changed over the treatment period, with a new collection of the *Bacteroidetes* lineages coming to dominate. Three *Helicobacter* OTUs reached significant enrichment in UIg (p<1×10^−11^ for two, p = 0.0036 for one). In the animals of group IIg, five Helicobacter OTUs were very significantly enriched (p<1×10^−10^ for two, p = 0.0009 for two, p = 0.01 for one), as well as one Lachnospiraceae OTU (p = 0.013).

The largest effects were seen after vancomycin treatment (IV group) in ceca from animals that either perished from the *C. difficile* infection or were euthanized when moribund. The luminal communities were not strongly affected, but the tissue-associated communities were very distinct, with much of the normal *Helicobacter* replaced by a *Bacteroides* lineage also found in the luminal contents. In addition to two *Helicobacter* OTUs (each p<0.001), substantially enriched amounts of *Clostridia* were detected in the vancomycin-treated tissue communities (p values between 0.014 and 2.4×10^−6^) as well as an *Anaerovorax* OTU (p<0.001). Thus, the data suggest that late stage disease diminished the distinction between the luminal community and the tissue-associated community in the animals of the IV group.

### Clindamycin treatment is associated with alterations of the microeukarya of the gut

**Figure 7:**
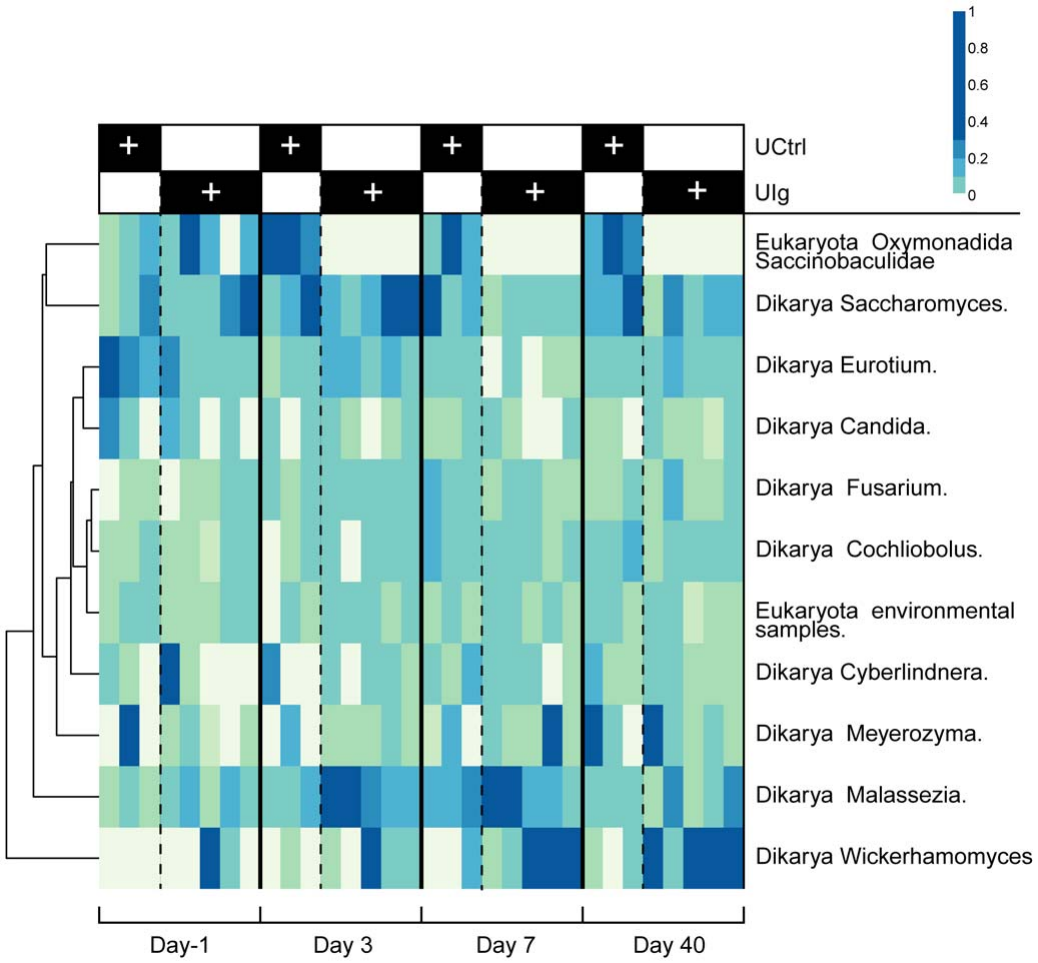
Taxonomic heatmap of 18S OTUs demonstrating microeukaryote succession in clindamycin-treated hamsters. Taxonomic heatmap comparing the three untreated control hamsters to five of the UIg individuals. The color code for the heat map is as in previous figures (more intense blue indicates a higher proportion).

We used 18S rRNA gene sequencing to probe the microeukaryal component of the intestinal microbiota, focusing initial characterization on the effects of clindamycin in isolation (Figure 7). Samples were taken over 40 days for three of the untreated control animals and five of the UIg group, and the effects of the antibiotic were assessed. DNA was amplified with the 18S_0067a_deg/NSR399 primer pair and initially worked up through the QIIME metagenomic analysis pipeline [28]. Taxonomic assignments were made using top BLAST hits as well as with BROCC, a pipeline for taxonomic assignment using voting on percent identity of the best BLAST hits [28]. The populations of single cell eukaryotes have not been studied extensively for hamster gut; therefore, sequence reads may derive from unstudied lineages, and so should be taken to indicate only the nearest group for which database sequences are available.

The microeukaryal communities were somewhat heterogeneous prior to treatment, and were rich in fungi, including members of *Candida*, *Saccharomycetaceae*, and *Trichocomaceae* (family containing *Aspergillus*/*Eurotium*). Three closely related, highly-abundant OTUs were called as possible chimeric sequences. When queried against the nr database from NCBI using blastn, the top results were *Saccinobaculus*, a poorly studied *Oxymonad*. While possibly artifactual, these data may instead correspond to an unidentified microeukaryote. The untreated community showed some variation over time, possibly reflecting the modest number of animals studied. Initially, unidentified fungi, in addition to *Trichocomaceae*, *Candida*, and the putative *Saccinobaculus*, were the major components of the community. The ultimate community was similar but altered by decreased numbers of *Trichocomaceae* and somewhat increased numbers of *Saccharomycetaceae* and unidentified fungi. The initial population for the clindamycin and mAb-treated hamsters was similar to that of the untreated control group. Following treatment with clindamycin and mAb, there was a temporary increase in *Mucorales*, an order containing notable opportunistic pathogens, and a more modest expansion of *Penicillium* and *Aspergillus*. Conversely, *Saccinobaculidae* and the *Entamoeba* (not shown), previously resident at a low abundance, disappeared after treatment. The antibiotic depletion of *Entamoeba* likely resulted from inhibition of the bacteria it consumes [29]. By day 40, the microeukarya returned to a community structure similar to the one present initially, though with elevated presence of some unidentified fungal OTUs and persisting absence of *Entamoeba* and *Saccinobaculus*. Thus, these data are, to our knowledge, the first high-throughput sequencing characterization of the hamster microeukarya and provide preliminary evidence that the fungal portion of the hamster microbiota is subject to succession associated with antibiotic treatment.

## Discussion

Here we present a longitudinal study of the effects of antibiotic treatment and subsequent *Clostridium difficile* infection in hamsters using deep sequencing. Our main finding was that bacterial communities showed considerable hysteresis; after perturbation with antibiotic treatment and *C. difficile* infection, the communities did not return to their initial states, but underwent several waves of succession and arrived at a new stable state.

The multiple waves of succession suggest a perturbed equilibrium where both antibiotic-sensitive bacteria and those reliant upon them decline, and species best able to respond rapidly to the new conditions thrive. As the antibiotic concentrations fall, species that grow more slowly or that are more sensitive begin to outcompete the early waves, suggested by *Proteobacteria* giving way to the *Bacteroidetes* and *Firmicutes* species. Factors such as encounter rate of potential colonists, niche specialization, and interaction with current inhabitants likely shape the species distribution until it reaches a stable composition with the available inhabitants [30]. This may be most evident in the *Bacteroidales* population, in which an ecological niche apparently selected for functionally similar residents during succession, but was limited by the availability of colonists to this alternate subset. These effects were remarkably consistent between the individually-housed hamsters placed in different rooms, which may be a result of the unusually homogenous microbiota shared by all of the animals at the outset. There is evidence that these replacement communities would shift if exposed to a more diverse set of colonists, such as that of a co-housed, antibiotic-naïve individual [31]. These findings suggest that antibiotic therapy could potentially be augmented by introducing a healthy complete microbiota while the microbiome is in this maleable state.

Several previous studies of antibiotic treatment in mice and humans have shown that the gut microbiota is profoundly and persistently altered by broad-spectrum antibiotic therapy [32] and arrives at a new stable state after treatment is withdrawn [31]. Culture-independent analyses of the effect of clindamycin in mice showed a broad suppression of *Bacteroidetes* and *Firmicutes* species, a large loss of diversity, and an ecological shift to more clindamycin-resistant taxa, notably the *Proteobacteria* species [33], [34], [35]. Reeves et al. also showed that susceptibility to *C. difficile* disease correlates with the extent of disruption of the gut microbiota through the use of varied antibiotic regimens and doses, perhaps as a result of specific but unknown changes to the structure of the community and not gross depopulation of the microbiome [34]. A separate study by Buffie et al. demonstrated a 90% loss of diversity in the intestinal communities of mice as a result of clindamycin administration, which led to a prolonged state of susceptibility to *C. difficile* despite near-complete community succession. The authors hypothesize that the protective subset of the microbiota, or members of the metabolic consortium on which they depend, may be particularly affected by clindamycin and slow to recover. [36]

A direct comparison between our work and the studies cited above cannot be readily made because of differences in the origin, type, and age of samples, as well as the different host species and recovery methods used. For example, whereas Buffie et al. studied samples extruded from the ilea and ceca of serially euthanized cohorts of mice, we analyzed longitudinal samples consisting of stool pellets recovered from bedding, excised cecal contents, and washed cecal tissue from individual hamsters. Despite these differences, we can draw some basic comparisons. While Buffie et al. report that two major groups, *Akkermansia* and *Mollicutes*, expanded in the wake of clindamycin therapy, these groups were mostly not present in the animals studied in our experiments. The third clade these authors observed was *Enterobacteriaceae*, a family in *Gammaproteobacteria* and closely related to the *Enterobacteriales*, *Burkholderiales*, and *Pasteurellales* that expanded in the hamsters after exposure to antibiotics. In contrast to the findings of Buffie et al., we found that the community effect of *C. difficile* infection on top of clindamycin administration to be modest. We cannot definitively explain this difference, but it is probable that host factors and initial community composition played a role. We also found that vancomycin had an effect on the microbiota, but the alteration induced by vancomycin treatment following clindamycin treatment was relatively small. Vancomycin also provided modest therapeutic benefits by temporarily suppressing *C. difficile*, presumably until drug levels fell below the minimum inhibitory concentration, at which point colonizing *C. difficile* grew out or environmental *C. difficile* was contracted. Similar to the observations by Buffie et al. in mice, we observed long-term carriage of *C. difficile* in clindamycin-treated hamsters with no frank disease. Stably-colonized hamsters could be the basis of an attractive alternative to the current relapse model of *C. difficile* in hamsters, in which infected hamsters are treated with a finite course of vancomycin and allowed to relapse as antibiotic suppression fades, ultimately producing outcomes similar to those observed in animals of the IV group reported in our studies. One recent clinical report states that as much as 87% of recurrent infections are a relapse more than 4 weeks after the initial episode and 65% after 8 weeks [36].

The sequencing data of the tissue-adherent bacterial community of the hamster cecum suggests that it is a low-diversity population of tissue colonization specialists, such as *Helicobacter* and possibly *Mucispirillum*, that are tolerated by the host. The significance of our findings of disrupted epithelial communities is unclear, but the binding of *C. difficile* to the mucosa appears to play a role in virulence and these organisms are well-placed to oppose that anchoring [37], [38], [39]. Furthermore, recent reports have shown that antimicrobial peptides are important for maintaining a protected buffer zone between the microbiota and the epithelial wall of the small intestine, which affects the activation of the adaptive immune system [40]. The findings of this study and others emphasize the presence of a dynamic tissue-associated community that may modulate pathogenesis [41].

A key question raised by our study is why the antibody treatment allowed the hamsters to survive even though the animals remained strongly positive for *C. difficile* by PCR in both the feces and cecum. In contrast, vancomycin treatment strongly reduced the load of *C. difficile* in stool, but ultimately failed to control the infection. In other studies, hamsters were consistently susceptible to as few as 100 spores [42]. Perhaps the antibodies persisted in sufficient quantities to inhibit toxin without further damage to the microbiota until sufficient suppressive microbes had grown out and rendered the *C. difficile* carriage benign. The pharmacokinetics and pharmacodynamics of the antitoxin antibodies are presently unknown, as is the anatomical site(s) associated with protection.. Perhaps the antibody treatment served as a protective bridge for the hamsters from the time of infection to the development of a protective adaptive immune response, involving the generation of endogenous, toxin-neutralizing antibodies. Further studies would be needed to clarify these or other potential mechanisms of action.

While the mAb treatment was capable of protecting the hamsters from death, it is interesting to note that the mAb-treated animals still appeared to suffer subclinical disease as evidenced by their weight loss post infection with *C. difficile*. The hamsters of group IIg were treated with anti-toxin mAbs before being infected with *C. difficile*; howeverthere is still some evidence that the animals of this group experienced effects of *C. difficile* infection as gauged by their initial loss of weight. Although non-toxigenic *C. difficile* is benign to the hosts, it has been shown that following infection of animals with a mixture of toxigenic and non-toxigenic strains in which toxin secretors formed less than 1% of the mixed community, infected animals were healthy, but had microscopic colitis [43]. This minor morbidity from a small number of toxin formers seems to reflect the results seen in this study, where even a relatively small amount of active toxin could also produce a non-lethal course of infection.

The pronounced and long-lived alterations in the gut *Oxymonad* and *Entamoeba* populations are a phenomenon that may be related to/associated with the reliance of *Oxymonads* and *Entamoeba* on symbiotic bacteria and prey bacteria, respectively [29], [44]. Furthermore, there are many established interactions between specific fungi and potential pathogens leading to enhanced virulence [45]. Moreover, *Saccharomyces boulardii* has been proposed as a probiotic therapy for CDAD as it seems to reduce toxin activity [46]. Much more research is required on this topic, but the infection model and sequencing tools presented here may provide new insight into these interactions.

In summary, we have shown that the intestinal microbiota of hamsters is initially homogenous and responds to and recovers from clindamycin perturbation in a predictable manner. This process involves multiple waves of succession and results in a final population of microorganisms in the clindamycin-treated animals that is distinct from the microorganism population in the untreated control community. Our findings show that neutralizing monoclonal antibodies directed against the toxins of *C. difficile* provide sufficient protection from toxin in the mAb-treated hamsters to allow their gut microbiota to stabilize. We also observed a loss of distinction between the luminal and the tissue-associated communities in the cecum, which may have significance for pathogenesis.

## Materials and Methods

### Ethics statement

To ensure humane treatment and minimize suffering, all animals were cared for with strict adherence to the protocol approved by the Ricerca IACUC committee under the guidelines set forth in the USDA Welfare Act (9 CFR Parts 1–3) and the “*Guide for Care and Use of Laboratory Animals*” (National Academy Press, Washington, DC, 1996).

### *C. difficile* culture and propagation

*C. difficile* strain ATCC 43596 was resuspended in 2× skim milk and stored at −80°C. This stock was streaked out on pre-reduced blood agar plates and allowed to incubate at 37°C for approximately 24 hours under anaerobic conditions. The bacteria were transferred to a new blood agar plate for repeat incubation. Colonies were inoculated into fluid thioglycolate medium (FTM) and grown for 24 hours as above. Bacteria were washed twice and resuspended in sterile phosphate-buffered saline [Biowhittaker, Basel, Switzerland]. The infectious cells were titered by dilution plate count to confirm a concentration of 4.2×10^6^ colony forming units per mL. All bacterial work was carried out under Biosafety Level 2 precautions.

### Antibiotic and mAb solution preparation

Clindamycin phosphate [C6427, Sigma-Aldrich Corp, St. Louis, MO] was dissolved in PBS to 5 mg/mL. Vancomycin [Sigma-Aldrich Corp] was dissolved in USP Purified Water to 2 mg/mL.

The *C. difficile* toxin-neutralizing monoclonal antibodies PA-50 and PA-41 were produced as described [17]. The antibody preparation was stored at −80°C immediately upon receipt and thawed at 2–8°C from day -2 to day -1. After dosing, the remaining solution was returned to a 2–8°C refrigerator until the next administration.

### Subject animals and care

Male Golden Syrian hamsters [*Mesocricetus auratu*s strain Crl:LVG(SYR), Charles River Laboratories, Inc., Stone Ridge, NY] included in the study were 70 days old and between 96–132 g, kinship unknown. Animals to be infected were diverted upon arrival to a separate room. They were individually housed in disposable plastic boxes [Innovive, Inc., San Diego, CA] on a rack with HEPA-filtered ventilation [Innovive, Inc.]. Uninfected hamsters were individually housed in polycarbonate boxes with filtered lids on stainless steel shelving. All animals were provided with food [2016 Teklad Global, Harlan, Indianapolis, IN] and tap water *ad libitum*. All hamsters received a health screen at enrollment and were allowed two days of acclimation prior to study start. Throughout the study, all cages were checked for dead or terminal animals daily. Due to weight loss observed in the mAb-treated groups, on veterinarian recommendation, all surviving hamsters on day 15 received 2 mL normal saline intraperitoneally. The animal care and experimentation were carried out at Ricerca Biosciences [Painesville, OH] in accordance with the protocol approved by Ricerca's IACUC Committee.

### Study design

Hamsters in the untreated control group received no interventions, while the animals in all other groups received a single subcutaneous injection of 50 mg/kg of clindamycin for sensitization on day -1 (Figure 1). Clindamycin-treated animals received antitoxin mAbs, vancomycin, or no treatment in either the absence (U0, UIg, UV) or presence (I0, IIg, IV) of inoculation with *C. difficile*. Inoculated animals received a 0.5 mL oral suspension of 4.2×10^6^ CFU/mL *C. difficile* ATCC 43596 on day 0. The groups receiving mAbs received 20 mg/kg/mAb intraperitoneally on days -1, 1, 3, and 5. From days 1 to 5, hamsters of the vancomycin treatment group received twice-daily oral doses of 20 mg/kg vancomycin. Based on findings of a prior study [17], intraperitoneal administration of 2 mL normal saline to all surviving animals was permitted if any exhibited excessive weight loss.

Stool samples were collected daily between days -1 and 3, every 2 to 3 days from day 5 to day 14, and weekly thereafter (Figure 1B). In addition, ceca were collected from all animals upon necropsy to sample the luminal and tissue-associated microbial populations (see example gross pathology in Figure 1C).

The day prior to all fecal sampling, the bedding of the uninfected hamsters was changed and both the cage and bedding of the infected hamsters were switched. Fecal samples were recovered from bedding daily from prior to clindamycin treatment on day -1 to day 3, approximately every other day thereafter until day 15, and approximately every 5 days until day 40. Ceca were removed at necropsy from all dead and euthanized, moribund hamsters, as well as from all remaining hamsters on day 40. All samples were frozen and stored at −80°C within one hour of recovery. Samples were shipped on dry ice and immediately stored in a −80°C freezer. Samples were analyzed for cultivable *C. difficile* and ribotype essentially as described [17] and [47]. The assay for cultivable *C. difficile* had a lower limit of detection of 100 CFU.

### DNA Extraction

Samples were processed with the PSP Spin Stool DNA Plus [1038110300, STRATEC Molecular, Berlin-Buch, Germany] kit with the following modifications. All extractions were carried out under BSL2+ conditions in a biological containment hood thoroughly cleaned with 10% bleach (0.615% w/v NaOCl). Approximately 110 mg of dry stool pellets, one stool tube scoop of cecal contents or all recoverable fluid from the cecal lumen, or a one cm segment of washed, opened distal cecal tissue were sterilely added to a pre-moistened Lysing Matrix E tube [116914100, MP Biomedicals, Solon, OH]. The tube was filled with stool stabilization buffer, followed by a five-minute incubation for dry pellets. Samples were homogenized for 81 seconds in a Mini-Beadbeater 16 [607, BioSpec, Bartlesville, OK] and incubated at 95°C for 15 minutes. Sample tubes were chilled on ice for one minute before centrifugation at 13,400× g for two minutes. All recoverable supernatant was transferred to InviAdsorb tubes with volume made up to 1.5 mL with stool stabilization buffer. The samples were vortexed for 15 seconds, incubated at room temperature for one minute, and then centrifuged at 16,000× g for three minutes. The supernatant was transferred to a new 1.5 mL tube and the centrifugation was repeated. From the tube of centrifuged InviAdsorb supernatant, 800 µL were added to 25 µL of proteinase K solution and incubated at 70°C. Binding buffer was added to the tubes in a 1∶2 ratio, mixed by pipetting, and 750 µL was transferred to a RTA spin filter column for a one minute incubation at room temperature. The columns were centrifuged at 9,300 rpm in a tabletop centrifuge for two minutes and transferred to a new RTA receiver tube. Each tube received 500 µL of Wash Buffer I, centrifuged for one minute at 9,300 rpm, and transferred to a new receiver tube. The columns were filled with 700 µL Wash Buffer II and centrifuged at 9,300 rpm for one minute. The flow through was discarded. To eliminate ethanol from Wash Buffer II, the filter was centrifuged for three minutes at 16,000× g. The elution was performed with 250 µL Elution Buffer D by centrifuging at 8,500× g. Eluted samples were stored at −20 C. Yield was generally 12.5–50 µg with A_260_/A_280_ of 1.90 or better.

### Amplification, sequencing, and sequence analysis

Sequences of all DNA primers used are in Supplementary Table 1. Amplification and sequencing of bacterial 16S rDNA genes were performed as previously described [48], with the following modifications. Dilutions of broad-specificity V1V2 primers and template, as well as quadruplicate 25 µL PCR reaction assembly, were carried out with a liquid-handling robot [epMotion 5075 LH, Eppendorf, Hamburg, Germany]. Reactions were performed by AccuPrime Taq DNA Polymerase [12339-016, Invitrogen, Carlsbad, CA], pooled and purified using AMPure magnetic beads [A63881, Agencourt, Brea, CA], and sequenced on a 454 Life Sciences FLX instrument [06372279001, Roche, Branford, CT] with Titanium reagents.

A *C. difficile*-specific Taqman qPCR assay [Applied Biosystems, Carlsbad, CA] was developed and carried out as previously described [41]. Primers employed were CD16S_102c_3′ and CD16S_239e_5′, paired with probe CD16S_198d_3′. Probe was conjugated with BHQ-1 [Biosearchtech, Novato, CA] and 6-fluorescein amidite (6-FAM).

Selective amplification of 18S genes was performed using primers 18S_0067a_deg [28] and NSR399 [49] with 12 base-pair barcodes were used to amplify the above DNA. The PCR cycle consisted of 5 minutes at 95°C for denaturing and polymerase activation, 35 cycles of 45 seconds at 95°C, 45 seconds at 56°C, and 90 seconds at 72°C, a single extension step of 72°C for 10 minutes, then maintained at 4°C. Remaining preparation was performed as above.

The QIIME pipeline was used for deep sequencing analysis [21]. Sequences were collapsed into consensus OTU sequences (cdhit at 97% identity) and classified using the RDP classifier (50% confidence threshold). Phylogenetic tree construction was performed using FastTree2. To test for relative enrichment between sample types, we first calculated a maximum likelihood estimate of the parameters of a Dirichlet-multinomial distribution using both samples. From this, we created a marginal Beta-binomial distribution function for each OTU that represented the null hypothesis of equal abundance in both sample types. P-values for enrichment were generated with a one-sided test against this marginal distribution function (Charlson et al., accepted with revisions). All analysis software is available at qiime.org and github.com/kylebittinger.

## Supporting Information

Table S1 **Sequences of oligonucleotides used in this study.**
(XLSX)
